# Different Somatic Hypermutation Levels among Antibody Subclasses Disclosed by a New Next-Generation Sequencing-Based Antibody Repertoire Analysis

**DOI:** 10.3389/fimmu.2017.00389

**Published:** 2017-05-03

**Authors:** Kazutaka Kitaura, Hiroshi Yamashita, Hitomi Ayabe, Tadasu Shini, Takaji Matsutani, Ryuji Suzuki

**Affiliations:** ^1^Repertoire Genesis Incorporation, Ibaraki, Japan; ^2^BITS Co. Ltd., Tokyo, Japan; ^3^Department of Rheumatology and Clinical Immunology, Clinical Research Center for Rheumatology and Allergy, Sagamihara National Hospital, National Hospital Organization, Sagamihara, Japan

**Keywords:** antibody, repertoire, sequencing, somatic hypermutation, class switch recombination

## Abstract

A diverse antibody repertoire is primarily generated by the rearrangement of V, D, and J genes and subsequent somatic hypermutation (SHM). Class-switch recombination (CSR) produces various isotypes and subclasses with different functional properties. Although antibody isotypes and subclasses are considered to be produced by both direct and sequential CSR, it is still not fully understood how SHMs accumulate during the process in which antibody subclasses are generated. Here, we developed a new next-generation sequencing (NGS)-based antibody repertoire analysis capable of identifying all antibody isotype and subclass genes and used it to examine the peripheral blood mononuclear cells of 12 healthy individuals. Using a total of 5,480,040 sequences, we compared percentage frequency of variable (V), junctional (J) sequence, and a combination of V and J, diversity, length, and amino acid compositions of CDR3, SHM, and shared clones in the IgM, IgD, IgG3, IgG1, IgG2, IgG4, IgA1, IgE, and IgA2 genes. The usage and diversity were similar among the immunoglobulin (Ig) subclasses. Clonally related sequences sharing identical V, D, J, and CDR3 amino acid sequences were frequently found within multiple Ig subclasses, especially between IgG1 and IgG2 or IgA1 and IgA2. SHM occurred most frequently in IgG4, while IgG3 genes were the least mutated among all IgG subclasses. The shared clones had almost the same SHM levels among Ig subclasses, while subclass-specific clones had different levels of SHM dependent on the genomic location. Given the sequential CSR, these results suggest that CSR occurs sequentially over multiple subclasses in the order corresponding to the genomic location of IGHCs, but CSR is likely to occur more quickly than SHMs accumulate within Ig genes under physiological conditions. NGS-based antibody repertoire analysis should provide critical information on how various antibodies are generated in the immune system.

## Introduction

B cells play a significant role in the adaptive immune system. They express B-cell receptor on their surface and produce its secreted form, antibodies, to neutralize antigens. Diversity of the antibody repertoire is essential to defend the body against the huge variety of potential foreign pathogens; it is primarily generated by recombination of the variable (V), diversity (D), and joining (J) gene segments and subsequently by somatic hypermutation (SHM), leading to the production of antibodies with optimized affinity for antigens. Class-switch recombination (CSR) generates nine antibody isotypes or subclasses (IgM, IgD, IgG3, IgG1, IgA1, IgG2, IgG4, IgE, and IgA2), which have different biological properties in the immune system (1), by replacing the proximal immunoglobulin (Ig) heavy constant (IGHC) gene with the distal IGHC. It has been reported that CSR occur primarily in a cytokine-dependent manner, for example, IL-4 induced IgG2 production in mice (2), IL-10 to IgG1 and IgG3 in human (3, 4), IL-4 to IgE in mice (5), and TGFβ to IgA in human (6). On the other hand, not only direct class-switching (IgM to IgE) but also multiple step sequential switch from IgM, IgA, IgG, to IgE have been reported (7).

Somatic hypermutations occur frequently across the V, D, and J regions of Ig genes, especially in complementarity-determining regions (CDRs) during affinity maturation (8–10). Both SHM and CSR share a common mechanism involving activation-induced cytidine deaminase (AID) (11). It is thus supposed that the rate of CSR varies from upstream Ig subclasses to downstream ones in a manner closely associated with SHM levels. Ig isotypes and subclasses were produced by both direct and sequential CSR in IgG (12), IgE (13–15), and IgA (16). According to a temporal model of IgE/IgG class switching (17), naive B cells initiate the switch from IgM/IgD to IgG3, then to IgG1 and to IgG2, and finally to IgG4 in the order corresponding to the genomic location of the IGHC genes. Sequential CSR is supposed to occur not only with a single antigen but also with consecutive antigen exposure in a secondary germinal center, into which primary IgG memory B cells re-enter (18). However, it remains poorly understood how antibody subclasses are generated and how many SHMs accumulate in respective subclasses. The SHMs that accumulate in Ig subclasses provide a significant measure not only of the extent of affinity maturation but also of how antibody subclasses are generated.

Next-generation sequencing (NGS) technologies capable of producing a huge amount of sequence information have improved dramatically, which has made antibody repertoire analysis essential for the identification of antigen-specific antibody genes, the diagnosis of B-cell lymphoma, and the evaluation of immune status following transplantation, vaccination, and infection as well as in autoimmune diseases (19, 20). Because SHMs frequently accumulate across antibody genes, long-read sequencing should be performed to comprehensively analyze SHMs. Among NGS platforms, an Illumina MiSeq platform produces 2 × 300-bp paired-end reads, allowing us to accurately determine nucleotide sequences including those of the VDJ and CDR3 regions of Ig segments and precisely analyze SHMs to characterize affinity maturation across all Ig genes. We have recently described an NGS-based T-cell receptor (TCR) repertoire analysis with an adaptor-ligation PCR (AL-PCR) method (21), which allows us to analyze immune repertoires more accurately than multiplex PCR. Thus, we applied the AL-PCR to amplify all antibody genes without bias and to determine their sequences with the discrimination of isotype and subclass genes. All transcripts from antibody genes were amplified separately for isotypes and commonly for subclasses with isotype-specific primers and a universal adaptor primer.

In this study, we revealed gene usage, diversity, SHM levels, and clonal sharing between Ig subclasses using peripheral blood mononuclear cells (PBMCs) from 12 healthy individuals. This study provides interesting findings on the difference in the SHM levels obtained with Ig clones shared or not shared among multiple antibody subclasses.

## Materials and Methods

### Peripheral Blood and RNA Extraction

All individuals did not have a disease history such as autoimmunity, allergy, or chronic infectious diseases. Ages of the individuals (11 males and 1 female) were 31.5 ± 11.8 (mean ± SD). Ten-milliliter aliquots of whole blood were collected into heparinized tubes. PBMCs were isolated using Ficoll-Paque™ (Pharmacia, Uppsala, Sweden) density-gradient centrifugation and washed with phosphate-buffered saline. The number of cells was counted and 1 × 10^6^ cells were used for RNA extraction. Total RNA was isolated from the PBMCs and purified with RNeasy Mini Kit (Qiagen, Hilden, Germany), in accordance with the manufacturer’s instructions. The amount and purity of RNA were measured using the Agilent 2100 Bioanalyzer (Agilent Technologies, Palo Alto, CA, USA).

### Unbiased Amplification of Antibody Genes with AL-PCR

One hundred nanograms of total RNA were converted to complementary DNA (cDNA) with Superscript III Reverse Transcriptase (Invitrogen, Carlsbad, CA, USA). BSL-18E primer containing polyT_18_ and a *Not*I site was used for cDNA synthesis. Following cDNA synthesis, double-stranded (ds)-cDNA was synthesized with *E. coli* DNA polymerase I (Invitrogen), *E. coli* DNA ligase (Invitrogen), and RNase H (Invitrogen), after which the ds-cDNA was blunted with T4 DNA polymerase (Invitrogen). P10EA/P20EA adaptor was ligated to the 5′ end of the ds-cDNA and then cut with the *Not*I restriction enzyme. After the removal of adaptor and primer with the MinElute Reaction Cleanup kit (Qiagen), PCR was performed with C-region-specific primers (heavy chain: IgM, IgD, IgG, IgA, and IgE) and P20EA (Table 1) with KAPA HiFi DNA Polymerase (Kapa Biosystems, Woburn, MA, USA). The PCR conditions were as follows: 95°C (20 s), 65°C (30 s), and 72°C (1 min) for 20 cycles. The second PCR was performed with C-region-specific nested primers and P20EA primers under the same PCR conditions. Amplicons were prepared by amplification of the second PCR products using P20EA-ST1 and C-region-specific Tag primer (Table 1). After PCR amplification, index (barcode) sequences were added by amplification with Nextera XT Index Kit v2 SetA (Illumina, San Diego, CA, USA). The indexed amplicon products were mixed in an equimolar concentration and quantified using a Qubit 2.0 Fluorometer (Thermo Fisher Scientific, Waltham, MA, USA). Sequencing was performed using the Illumina Miseq paired-end platform (2 × 300 bp).

**Table 1:** **Primers used in this study**.

Primer	Sequence
BSL-18E	AAAGCGGCCGCATGCTTTTTTTTTTTTTTTTTTVN
P20EA	TAATACGACTCCGAATTCCC
P10EA	GGGAATTCGG
P22EA-ST1-R	GTCTCGTGGGCTCGGAGATGTGTATAAGAGACAGCTAATACGACTCCGAATTCCC
CM1	TGATGTCAGAGTTGTTCTTG
CM2	TCCTGTGCGAGGCAGCCAA
CM-ST1-R	TCGTCGGCAGCGTCAGATGTGTATAAGAGACAGGTATCCGACGGGGAATTCTC
CG1	CACCTTGGTGTTGCTGGGCTT
CG2	TCCTGAGGACTGTAGGACAGC
CG-ST1-R	TCGTCGGCAGCGTCAGATGTGTATAAGAGACAGTGAGTTCCACGACACCGTCAC
CA1	GCTGGCTGCTCGTGGTGTAC
CA2	GGGAAGTTTCTGGCGGTCACG
CA-ST1-R	TCGTCGGCAGCGTCAGATGTGTATAAGAGACAGGGGGAAGAAGCCCTGGACCA
CD1	GTCCCGTCTTTGTATCTCAG
CD2	TCTGTGTCCCCATGTACC
CD-ST1-R	TCGTCGGCAGCGTCAGATGTGTATAAGAGACAGCCCAGTTATCAAGCATGCC
CE1	CATAGTGACCAGAGAGCGTG
CE2	GGTGGCTGGTAAGGTCATA
CE-ST1-R	TCGTCGGCAGCGTCAGATGTGTATAAGAGACAGCATTGGAGGGAATGTTTTTG

### Assignment of Ig Heavy Chain Sequences

The identification of V, D, J, and C regions was performed by determining the sequence with the highest identity to reference sequence data sets available from the international ImMunoGeneTics information system^®^ (IMGT) database (http://www.imgt.org). Nucleotide sequences from 5′ adaptor primer to IgH C region were determined and used for further repertoire analysis. The 5′ terminal sequence is somewhat partial and homogenous but contain almost FR1 to CDR3 of IgH sequences. The data processing, assignment, and data aggregation were automatically performed using repertoire analysis software developed in-house (Repertoire Genesis™). The RG implemented a program for sequence homology search using BLASTN, an automatic aggregation program and a graphics program for V usage, J usage, and CDR3 length distribution. CDR3 sequences were defined as sequences ranging from conserved cysteine (Cys104) at position 104 of the IMGT nomenclature to conserved tryptophan (Trp118) or phenylalanine (Phe118) at position 118.

### Data Analyses and Diversity Index

The identical V, D, J, and deduced amino acid sequences of CDR3 were defined as a unique sequence read (USD). A USD contains several variant sequences formed by SHMs that occur frequently during antibody maturation. Therefore, we consider sequence reads sharing identical V, D, and J segments and identical amino acid sequences of CDR3 as being from an identical clonal lineage. The numbers of USDs were automatically counted using Repertoire Genesis software and eventually ranked in order of copy number. To estimate the repertoire diversity in respective isotypes and subclasses, a diversity index, the Shannon–Weaver index, was calculated using the “diversity” function of the vegan package in the R program. The Shannon–Weaver index was normalized by dividing by the logarithm of the total number of unique reads, ln(*N*).

### Analyses of CDR3 Length and Amino Acid Composition

The deduced amino acid sequence of CDR3 was used for further analysis of length and amino acid composition. The number of in-frame reads with each CDR3 length (0–40 amino acids) was counted. To demonstrate alteration of the CDR3 length, difference in the percentage of CDR3 length between subclasses were calculated. Position of amino acid in CDR3 sequence was classified according to CDR3-IMGT nomenclature. Amino acid composition of each position from anchored Cys104 to Trp118 or Phe118 was calculated. Physicochemical properties were calculated using the peptide package in the R program.

### Statistics

Statistical significance was tested with a non-parametric Kruskal–Wallis test with Dunn’s multiple comparisons posttest to compare three or more unpaired groups. The Mann–Whitney test was used to analyze the difference between two groups. The statistical tests were performed using GraphPad Prism software (San Diego, CA, USA).

## Results

### NGS of Nine Classes of IgH Genes

**Figure 1:**
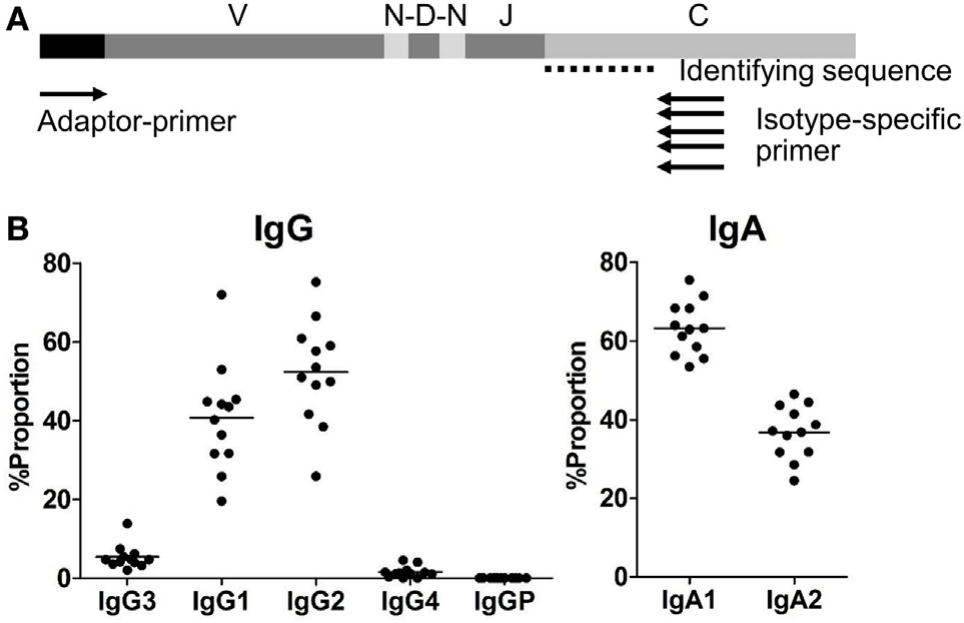
**(A)** Adaptor-ligation PCR for next-generation sequencing-based antibody repertoire analysis. A universal primer specific for the isotype constant region (isotype-specific primer) and an adaptor primer were used for unbiased amplification of antibody genes. IgM-, IgD-, IgG-, IgA-, and IgE-specific primers were used for amplification of respective isotype genes. Amplified gene products were sequenced using an Illumina MiSeq sequencer, and each sequence read was classified into immunoglobulin subclasses by discrimination using the identifying sequence of the constant region. **(B)** Proportions of IgG and IgA subclasses in healthy individuals. Proportions of sequence reads of IgG subclasses (IgG3, IgG1, IgG2, IgG4, and IgGP) and IgA subclasses (IgA1 and IgA2) are indicated. Total sequence reads excluding unproductive reads (out-of-frame reads) were used for calculation. Each dot represents an individual and bars indicate mean frequencies (*n* = 12).

We performed NGS analyses of IgM, IgG (four subclasses: IgG1, IgG2, IgG3, and IgG4), IgA (two subclasses: IgA1 and IgA2), IgD, and IgE using the Miseq Illumina platform technologies (Table 1). The C-region-specific primers were designed to amplify five isotype genes (IgM, IgD, IgG, IgA, and IgE) and identity IgG subclasses (IgG1, IgG2, IgG3, and IgG4) and IgA subclasses (IgA1 and IgA2) by determining the identifying sequence within the amplified sequences (Figure 1A). After filtering for quality, a total of 5,480,040 sequence reads were obtained from the PBMCs of 12 healthy individuals (Table S1 in Supplementary Material). The sequence reads were compared with IGHV, IGHD, IGHJ, and IGHC reference sequences available from the IMGT website (http://www.imgt.org/). V, D, J, and C regions were automatically assigned using an original program developed in-house called Repertoire Genesis. Unproductive reads (out-of-frame reads) carrying a stop codon or a shift of reading frame in the CDR3 region were excluded and the remaining 5,353,863 in-frame sequence reads were used for further analysis (Table S2 in Supplementary Material). To evaluate the accuracy and quality of the Miseq sequencing, we calculated the frequency of mismatch in the C region of Ig genes between query and reference sequences as the error rate. The error rates were 0.14% (IgM), 0.10% (IgD), 0.18% (IgG), 0.21% (IgA), and 0.37% (IgE). These rates were low enough to evaluate SHM in Ig genes.

### Proportions, Diversity, and Usages in IGH Subclasses

**Figure 2:**
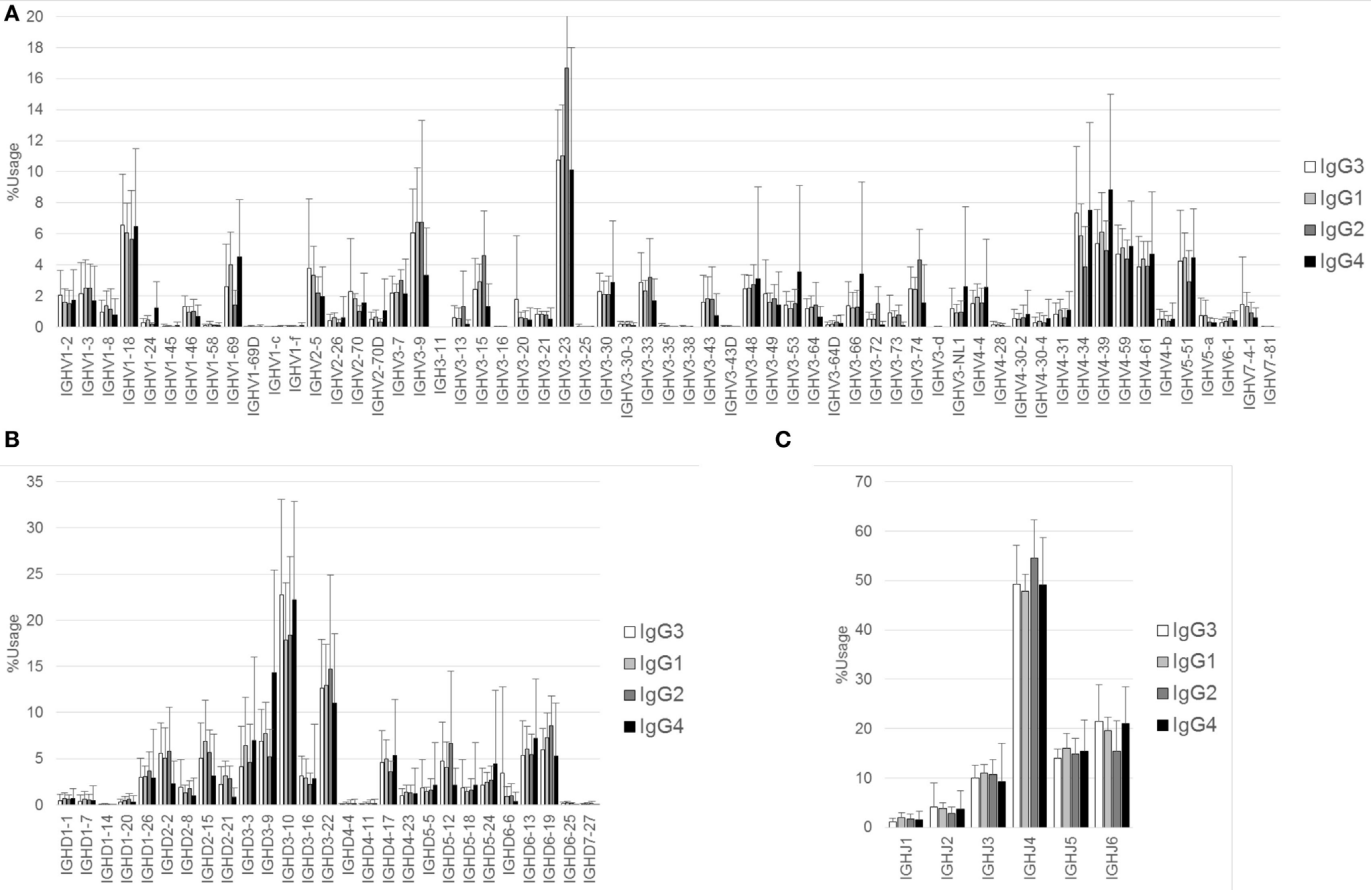
**Comparison of usage of IGHV, IGHD, and IGHJ among IgG subclasses**. Mean percentage usages of IGHV **(A)**, IGHD **(B)**, and IGHJ **(C)** in IgG3, IgG1, IgG2, and IgG4 are shown. Bars and error bars indicate mean percentage usage and its SD of 12 healthy individuals, respectively. Overall, there were several small differences in the usages of IGHV, IGHD, and IGHJ among IgG subclasses. For IGHV, significant differences were found in IgG3 vs. IgG2 (IGHV3–23, *P* < 0.01; IGHV4–34, *P* < 0.001), IgG3 vs. IgG4 (IGHV3–9, *P* < 0.01; IGHV4–39, *P* < 0.001), IgG1 vs. IgG2 (IGHV1–69, *P* < 0.05; IGHV3–23, *P* < 0.001), IgG1 vs. IgG4 (IGHV3–9, *P* < 0.001; IGHV3–53, *P* < 0.05; IGHV4–39, *P* < 0.01), and IgG2 vs. IgG4 (IGHV1–69, *P* < 0.001; IGHV3–9, *P* < 0.001; IGHV3–15, *P* < 0.001; IGHV3–23, *P* < 0.001; IGHV3–74, *P* < 0.01; IGHV4–34, *P* < 0.001; IGHV4–39, *P* < 0.001). For IGHD, significant differences were found in IgG3 vs. IgG1 (IGHD3–10, *P* < 0.05), IgG3 vs. IgG4 (IGHD3–9, *P* < 0.001), IgG1 vs. IgG4 (IGHD3–9, *P* < 0.001), and IgG2 vs. IgG4 (IGHD3–9, *P* < 0.001; IGHD5–12, *P* < 0.05). For IGHJ, significant differences were found in IgG3 vs. IgG2 (IGHJ4, *P* < 0.05; IGHJ6, *P* < 0.05), IgG1 vs. IgG2 (IGHJ4, *P* < 0.01), and IgG2 vs. IgG4 (IGHJ4, *P* < 0.05; IGHJ6, *P* < 0.05).

**Figure 3:**
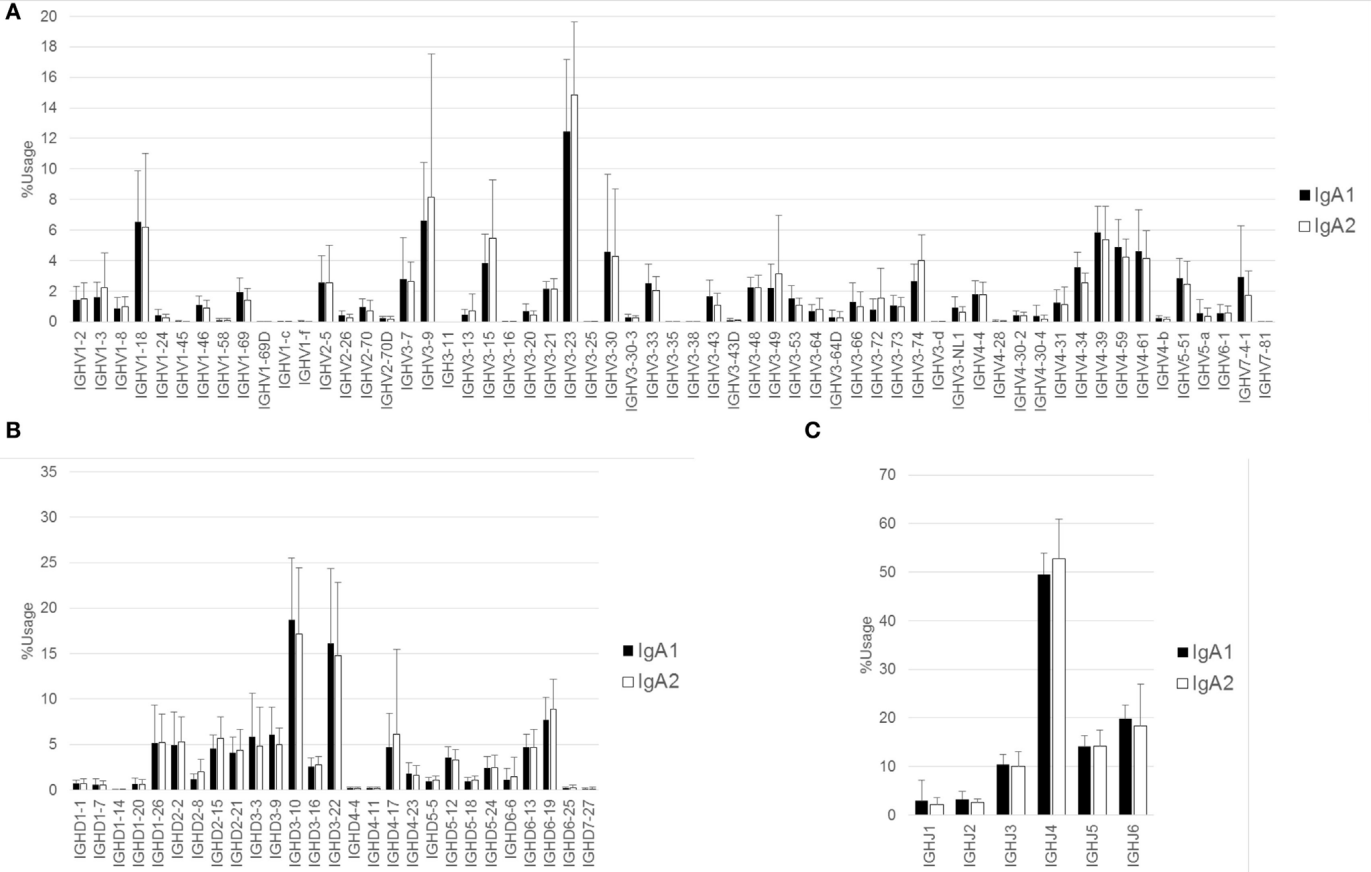
**Comparison of usage of IGHV, IGHD, and IGHJ among IgA subclasses**. Mean percentage usages of IGHV **(A)**, IGHD **(B)**, and IGHJ **(C)** were compared between IgA1 and IgA2. Bars and error bars indicate mean percentage usage and SD of 12 healthy individuals, respectively. There were significant differences in IGHV3–35 (*P* < 0.001), IGHV3–74 (*P* < 0.05), and IGHV4–34 (*P* < 0.01).

The proportions of IgG and IgA subclasses were calculated by counting in-frame sequence reads matched with subclass-specific sequences (identifying sequences) (Figure 1B). The proportions of IgG1 (mean ± SD: 40.7 ± 13.7%) and IgG2 (52.4 ± 13.1%) were higher than those of IgG3 (5.3 ± 3.0%) and IgG4 (1.5 ± 1.4%). There was no significant difference in the proportions between IgG1 and IgG2. The proportion of IgA1 was significantly higher than that of IgA2 (63.2 ± 6.7 vs. 36.8 ± 6.7%, *P* < 0.0001). Diversity of the antibody repertoire among Ig subclasses was evaluated using the Shannon–Weaver diversity index normalized to exclude the effect of read number. There were no significant differences in the diversity among the Ig subclasses other than IgE, which had lower diversity than the others (Figure S1 in Supplementary Material). To clarify differences in the usage of IGHV, IGHD, and IGHJ among the Ig subclasses, the frequencies of IGHV, IGHD, and IGHJ genes were compared among the Ig subclasses (Figures 2 and 3). For the IgG subclasses, there were no differences in the usage of IGHV, IGHD, and IGHJ among the IgG3, IgG1, IgG2, and IgG4 subclasses. These results indicated that CSR did not impact on the usage of IGHV, IGHJ, and IGHD repertoires.

### CDR3 Length and Amino Acid Composition of IgH Subclasses

**Figure 4:**
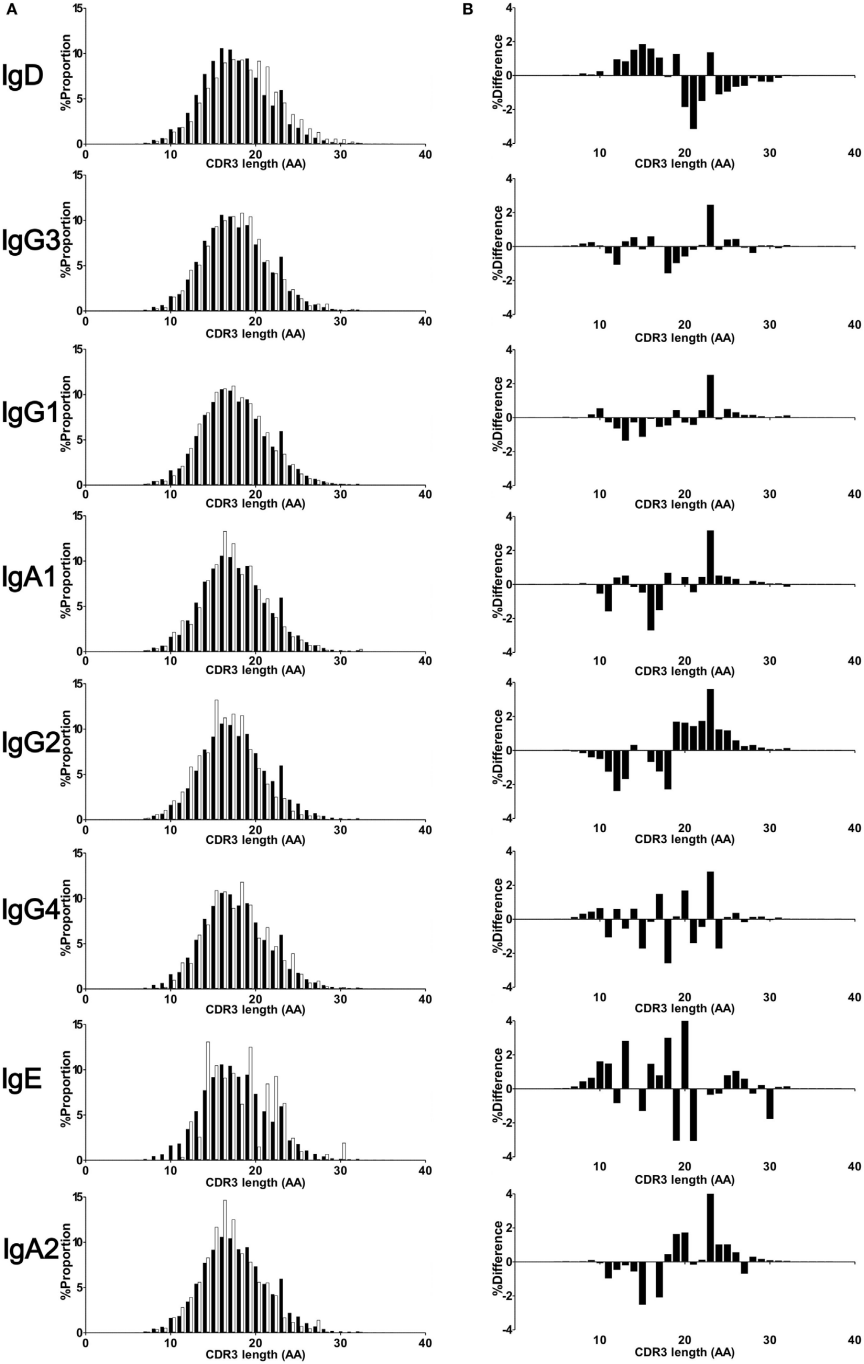
**CDR3 length of isotypes and subclasses**. Deduced amino acid sequence of CDR3 region of IgM, IgD, IgG3, IgG1, IgA1, IgG2, IgG4, IgE, and IgA2 were collected from 12 individuals. The CDR3 length was calculated based on amino acid sequence from conserved Cys104 to Trp118 or Phe118. Percentage frequencies of each CDR3 length were plotted by a bar plot **(A)**. Closed bar indicates percentage frequencies in CDR3 length of IgM (control), while open bar indicates respective immunoglobulin subclasses. To compare difference in the CDR3 length between immature and mature B cells, difference in the percentage frequencies between IgM and either IgD, IgG, IgA, and IgE or IgM were plotted at each position **(B)**. Negative values in the left half and positive values in right half indicate a shortening of CDR3 length in respective Igs compared with IgM.

Amino acid length of CDR3 was calculated in respective Ig isotypes and subclasses. All isotypes and subclasses showed Gaussian-like distributions with multiple peaks (Figure 4). Median length of CDR3 were 17 (IgM), 19 (IgD), 18 (IgG), 17 (IgA), and 18 (IgE), respectively. The length was longer in IgD than in IgM (mean ± SD, IgD: 18.8 ± 4.1, IgM: 18.0 ± 3.9, *P* < 0.001), while IgG, IgA, and IgE showed shorter length compared with IgM (IgG: 17.9 ± 3.8, IgA: 17.6 ± 3.6, IgE: 17.6 ± 3.6, all *P* < 0.001). To demonstrate alteration of CDR3 length between immature and mature B cells, difference in the percentage frequencies of CDR3 length of IgD, IgG, IgA, and IgE from IgM were plotted at each position. CDR3 length shortened during the process from IgM to IgG, IgA, and IgE subclasses. This was a straightforward illustration showing CDR3 shortened during the process of Ig class-switch. This feature seems to be similar with the selection of shorter TCR during T cell development. To examine alteration of CDR3 amino acid composition, percentage frequencies of 20 amino acid was calculated at each position of CDR3 (Figure S2 in Supplementary Material). The result showed a highly restricted usage in anchored position 104 (Cys) and 118 (Tryp), and a relatively restricted usage in position 105 (Ala), 106 (Arg), 116 (Asp), and 117 (Tyr). As a whole, there was a similar amino acid composition at the other positions among Ig isotypes and subclasses. Furthermore, in order to examine difference in the physicochemical properties of CDR3 amino acid sequence, proportions of amino acid classified by structural and functional properties were compared among Ig subclasses (Figure S3 in Supplementary Material). The result indicated that the composition of functional residues were relatively similar among IgM, IgG3, IgG1, IgA1, and IgG3. Notably, IgG4 showed higher proportion of basic, charged, and polar residues but lower proportion of non-polar and aromatic in comparison with IgM, indicating IgG4 has characteristics of amino acid composition slightly different from the other subclasses.

### SHM in Ig Subclasses

**Figure 5:**
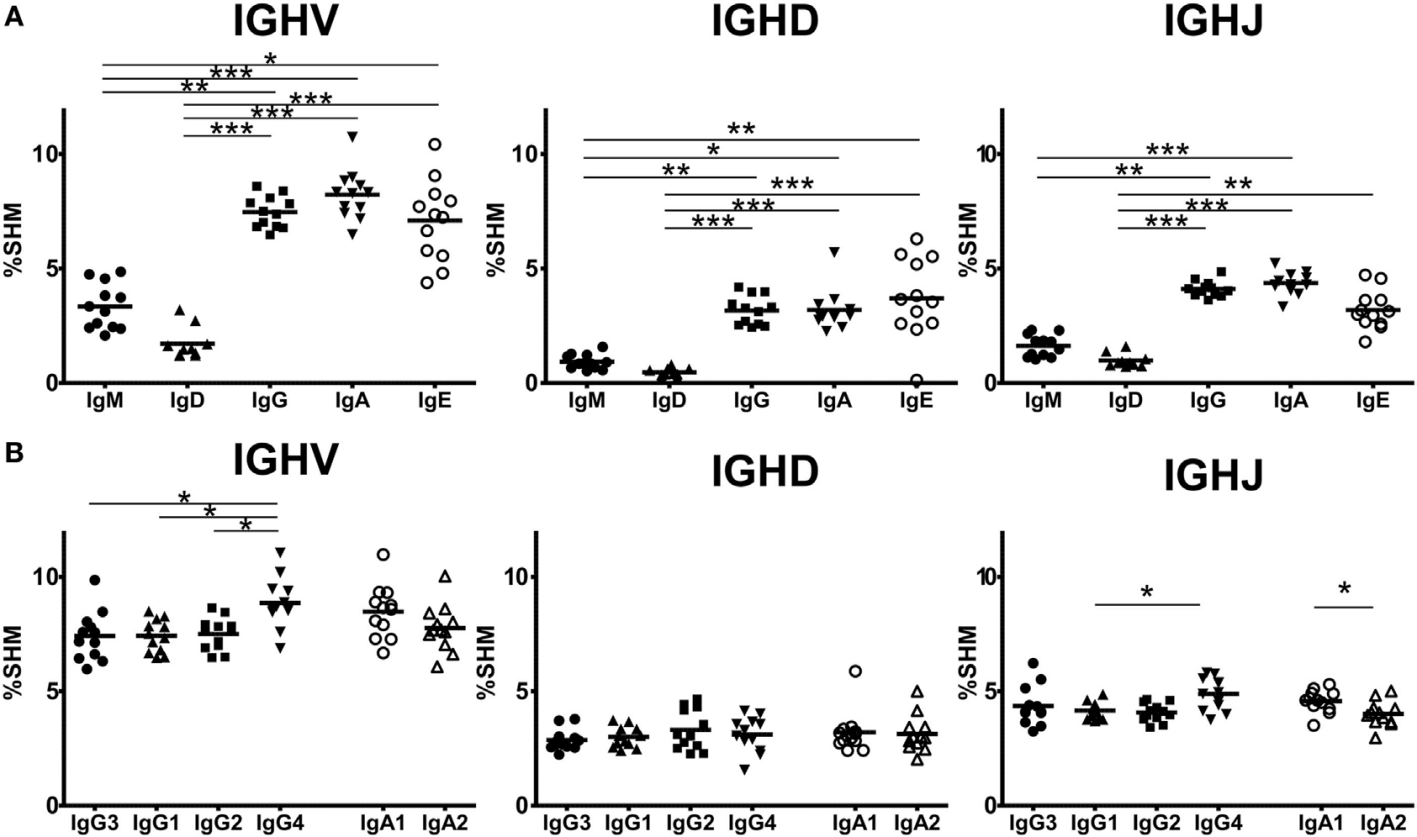
**Somatic hypermutation (SHM) levels in immunoglobulin isotypes and subclasses**. Frequencies of SHM in isotypes **(A)** and IgG and IgA subclasses **(B)** are shown. Frequencies of SHM in variable region (IGHV), diversity region (IGHD), and joining region (IGHJ) were calculated by comparison of sequence reads with the reference sequence. Sequence data from in-frame IgM (1,338,009 reads), IgD (1,279,881), IgG3 (60,000), IgG1 (503,101), IgG2 (667,076), IgG4 (19,708), IgA1 (866,180), IgA2 (519,390), and IgE (87,474 reads) were used to calculate SHM. For IGHV and IGHD, the SHM levels of IgG, IgA, and IgE were significantly higher than those of IgM and IgD. Similarly, for IGHJ, the SHM levels of IgG and IgA were significantly higher than those of IgM and IgD and the SM levels of IgE were significantly higher than those of IgD. Regarding IgG and IgA subclasses, IgG4 had significantly higher SHM levels in IGHV regions than IgG3, IgG1, and IgG2 (*P* < 0.05). The SHM levels of IgG4 were significantly higher than those of IgG1. On the other hand, there were no significant differences in the SHM levels in both IGHV and IGHD between IgA1 and IgA2, but the SHM levels of IgA2 was significantly lower than those of IgA1. Significance: *P < 0.05, **P < 0.01, and ***P < 0.001.

Somatic hypermutation levels were compared for IGHV, IGHD, and IGHJ among the four Ig isotypes by comparing sequences bearing isotype IGHC with reference sequences (Figure 5A). IgG, IgA, and IgE had more frequent SHM than immature IgM and IgD (IgM: 3.34 ± 0.99%, IgD: 1.73 ± 0.69%, IgG: 7.47 ± 0.69%, IgA: 8.22 ± 1.08%, IgE: 7.01 ± 1.77%). However, there were no differences in the SHM levels between IgM and IgD or among IgG, IgA, and IgE. These results indicated that class-switched Ig isotypes had higher SHM levels than IgM/IgD, but their SHM did not vary among isotypes. Next, to examine whether SHM levels differed among IgG or IgA subclasses, these levels were compared for the IGHV, IGHJ, and IGHJ regions among IgG3, IgG1, IgG2, and IgG4 or between IgA1 and IgA2 (Figure 5B). Overall, SHM occurred most frequently in IGHV regions, but moderately in IGHD and IGHJ. For IGHV sequences, the SHM levels were significantly higher in IgG4 than in the other IgG subclasses (IgG3: 7.14 ± 1.07%, IgG1: 7.42 ± 0.71%, IgG2: 7.50 ± 0.70%, IgG4: 8.86 ± 1.09%, IgG1 vs. IgG4 and IgG2 vs. IgG4: *P* < 0.05, IgG3 vs. IgG4: *P* < 0.01). There was no difference in the SHM levels in IGHD and IGHJ among IgG subclasses. However, SHM was significantly higher in IgA1 than in IgA2 (IgA1: 8.48 ± 1.15%, IgA2: 7.76 ± 1.01%, *P* < 0.05), while there was no significant difference in the SHM levels of IGHD and IGHJ.

### Rate of Sequence Sharing among Ig Subclasses

**Figure 6:**
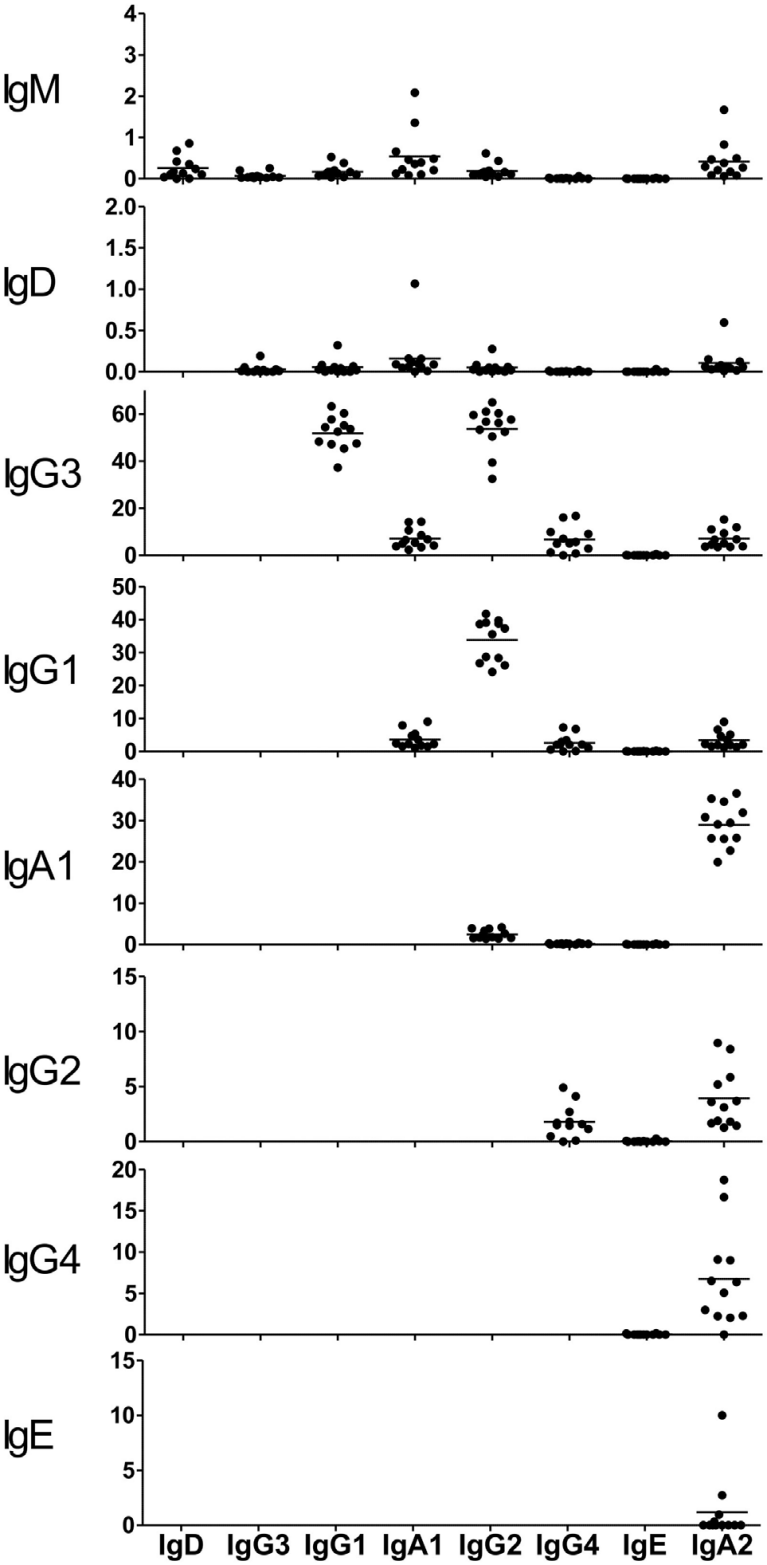
**Clone sharing among immunoglobulin (Ig) isotypes and subclasses**. The proportions of clones shared between two subclasses are shown. Each dot represents an individual. Sequence reads sharing identical V, D, and J segments and identical amino acid sequences of CDR3 were considered as being from identical clonal lineages. The proportions were calculated by dividing the number of shared clones by the total number of ancestral Ig (upstream). Each Ig isotype and subclass is shown in the order corresponding to the genomic location of IGHC regions (22).

Class-switch recombination occurred first at IgM/IgD and then at downstream Ig subclasses in the order corresponding to the genomic location of the C-region genes. To explore which Ig subclasses are derived from which other Ig subclasses, the rate of clonal lineage sharing between two subclasses was examined (Figure 6). Sequence reads sharing identical V, D, and J segments and identical amino acid sequences of CDR3 were considered as being from identical clonal lineages. IgM and IgD were poorly shared with IgG subclasses, instead being more frequently shared with IgA1 and IgA2 subclasses. The results showed that class switching from IgM to either IgA1 or IgA2 is the typical type. The proportion of sequences shared between IgM and IgG3 as seen in IgM was around 0.1%, indicating that only a few IgM^+^ cells switch to IgG3. Class switching of IgG3 to IgG1 or IgG2 occurs more frequently than the other types, and the efficiency of switching from IgG3 to IgG1 was almost the same as that to IgG2 (51.9 vs. 53.7%). A small proportion of IgG3 was shared with not only IgG4 but also IgA1 and IgA2 subclasses (6–7%), indicating that CSR normally occurs from IgG to IgA. Sequence reads were highly shared between IgG1 and IgG2 (33.8%) and between IgA1 and IgA2 (29.0%). This indicates that class switching occurs more efficiently from IgG1 to IgG2 or from IgA1 to IgA2. In addition, class switching from IgG2 to IgA2 (3.9%), from IgG2 to IgG4 (1.8%), and from IgG4 to IgA2 (6.8%) normally occurs in healthy individuals.

### Sequence Sharing over Multiple Ig Subclasses

To examine how isotype or subclass antibodies are generated from immature ones, a search for the sharing of identical sequences of VDJ and CDR3 was conducted, which was counted in all data sets of the subclasses (Table S3 in Supplementary Material). A majority of clones were found in only a single Ig subclass, and those in IgM and IgD were almost all isotype-specific. Ig clones shared between IgA1 and IgA2 and between IgG1 and IgG2 were abundant, and sequence reads shared among multiple subclasses were occasionally observed in the data sets. For example, IgG3–IgG1–IgG2 (6,789, 0.78%), IgG3–IgG1–IgG2–IgG4 (816, 0.09%), and IgG1–IgG2–IgG4 (770, 0.09%) shared clones were observed. Interestingly, IgG3–IgG1–IgA1–IgG2–IgA2 (782, 0.09%), IgA1–IgG2–IgA2 (544, 0.06%), IgG1–IgA1–IgG2 (431, 0.05%), and IgG1–IgG2–IgA2 (384, 0.04%) shared clones were also found. These results indicated that a significant number of clones divergent from identical clonal lineages existed in multiple Ig subclasses.

### Relationship of SHM with CSR

**Figure 7:**
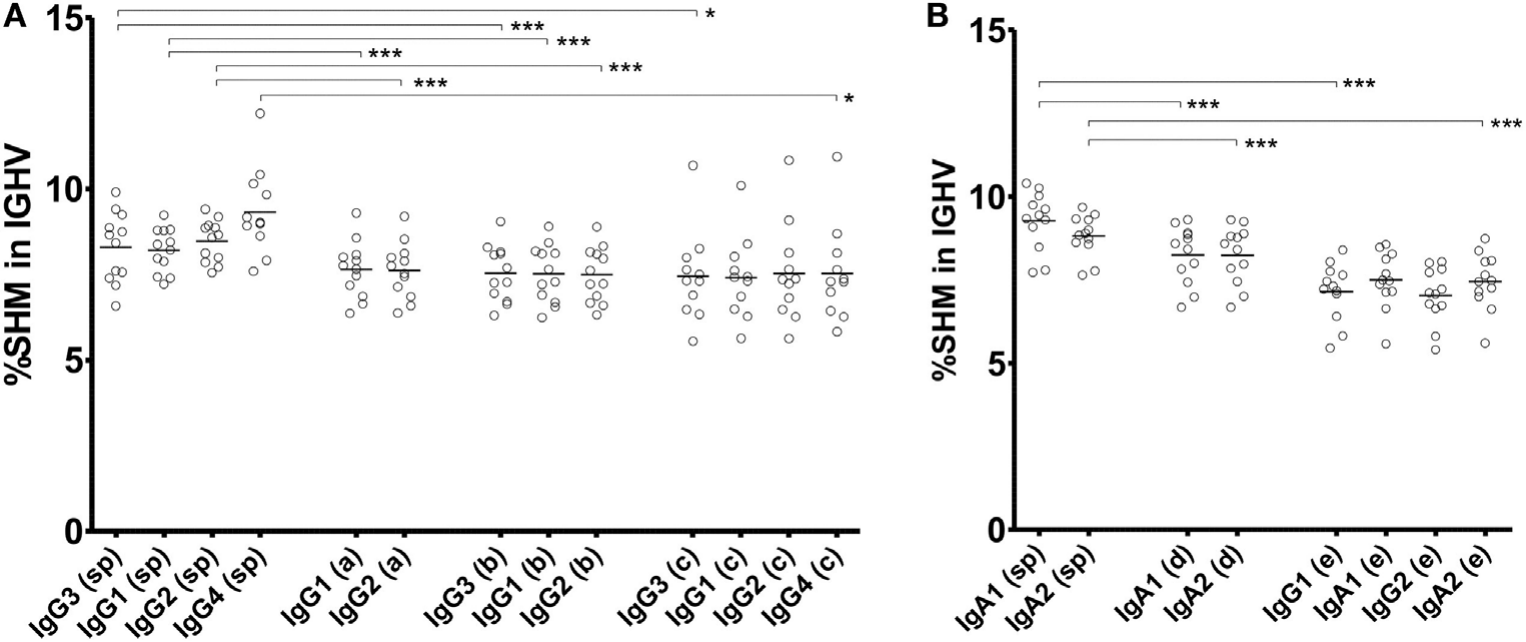
**Somatic hypermutation (SHM) levels of subclass-specific and shared clones in IgG (A) and IgA (B) subclasses**. The frequencies of SHM within the immunoglobulin (Ig) variable region (IGHV) in subclass-specific clones and clones shared among multiple subclasses are shown. Sequence reads found in only an Ig subclass were considered as subclass-specific clones (sp). SHM levels in sequence reads shared between IgG1 and IgG2 (double, a); among IgG3, IgG1 and IgG2 (triple, b); among IgG3, IgG1, IgG2, and IgG4 (quadruple, c); between IgA1 and IgA2 (double, d); and among IgG1, IgA1, IgG2, and IgA2 (quadruple, e) were compared. Statistical significance was tested by Wilcoxon rank-sum test (*P < 0.05, **P < 0.01, and ***P < 0.001).

There were many Ig clones sharing VJ and CDR3 within multiple Ig subclasses. If these Ig clones switched from an ancestral subclass to a descendant one, then SHMs accumulated more frequently in the descendant subclass. Thus, SHM levels were examined in individual clones shared among multiple Ig subclasses (Figure 7). Most clones were detected only within subclasses (subclass-specific), which had different levels of SHM. IgG4 showed the highest levels of SHM among the IgG subclasses, while similar levels of SHM were observed in IgG3, IgG1, and IgG2. Clones shared between IgG1 and IgG2 had lower SHM levels than IgG1- or IgG2-specific clones. Similarly, clones shared between IgA1 and IgA2 had lower levels than IgA1- or IgA2-specific clones. The SHM levels of IgG1-specific clones were significantly higher than those of clones shared between IgG1 and IgG2 in the IgG1 subclasses (IgG1-specific: 7.64 ± 0.83%, IgG1-shared: 7.52 ± 0.84%, *P* < 0.001; Wilcoxon rank-sum test). Similarly, IgG2-specific clones had higher SHM than IgG2-shared clones (IgG2-specific: 7.61 ± 0.81%, IgG2-shared: 7.49 ± 0.80%, *P* < 0.001). Considering that these shared clones had just switched between them, SHMs occurred at a low level in them, but accumulated as time passed after the switching. Interestingly, clones shared among three or more subclasses had similar levels of SHM to each other, while IgG4 never had higher levels of SHM than the others. The SHM levels were significantly higher in IgG4-specific clones than in IgG4 clones shared among multiple subclasses (IgG4-specific: 9.32 ± 1.22%, IgG4-shared of IgG3–IgG1–IgG2–IgG: 7.53 ± 1.40%, *P* < 0.001; IgG4-shared of IgG1–IgG2–IgG: 6.99 ± 1.20%, *P* < 0.001). These results indicated that CSR occurred sequentially over multiple subclasses without the accumulation of SHMs. IgA1-specific and IgA2-specific clones had higher SHM levels than IgA1–IgA2-shared clones in IgA1 and IgA2 subclasses, respectively (IgA1-specific: 9.27 ± 0.88%, IgA1-shared: 8.24 ± 0.87%, *P* < 0.001; IgA2-specific: 8.82 ± 0.25%, IgA2-shared: 8.24 ± 0.86%, *P* < 0.001). Remarkably, clones shared among IgG1, IgA1, IgG2, and IgA2 had different levels of SHM between IgG and IgA. The shared clones presented in IgA1 and IgA2 showed moderate levels of SHM and their SHM levels were higher than those of IgG1 and IgG2 (IgG1: 7.14 ± 0.87%, IgA1: 7.49 ± 0.84%, IgG2: 7.02 ± 0.83%, and IgA2: 7.45 ± 0.84%).

## Discussion

Large-scale NGS of antibodies is a powerful tool to obtain a better understanding of humoral immunity in physiological and pathological conditions. Unlike in TCR genes, SHM occurs frequently in Ig genes during the maturation of B cells, by which antibodies acquire higher affinity to target antigens. In the case of multiplex PCR using V-region-specific and J-region-specific primers, amplification bias is inevitable because of nucleotide mismatches between a target sequence changed by SHM and a primer designed based on the germline reference sequence. Instead, adaptor-ligation PCR, one of the RACE techniques, is capable of amplifying all Ig genes without PCR bias, allowing us to accurately quantify individual Ig genes and further evaluate SHM levels in individual Ig subclasses.

Class-switch recombination is capable of generating various antibodies having the same antigen specificity but different isotypes or subclasses. These isotypes or subclasses have different biological properties, functional locations, and abilities to deal with different antigens. IgG subclasses differ in structure, Fcγ receptor binding and immune complex formation with complement (1, 23). Abnormal production of certain Ig subclasses is reportedly associated with diseases such as autoimmunity (24, 25). Many studies on the accumulation of SHMs in antigen-specific B cells after vaccination have also been reported. In this study, we provided important information on subclass distribution, affinity maturation, and the CSR process in Ig genes of healthy individuals under physiological conditions. This constitutes control data that are essential for determining antibody abnormalities in infected or diseased individuals.

We observed similar subclass compositions of IgG and IgA in healthy individuals: IgG1 and IgG2 were abundant, IgG4 was the rarest of all IgG subclasses, and IgA1 had a higher proportion than IgA2. These results are well consistent with the serological distribution (26), indicating that the serum levels of antibodies are closely correlated with the expression levels of subclasses. We did not find any differences in the usage of IGHV, IGHD, and IGHJ among the Ig subclasses. In addition, diversity did not change during the switching from the ancestral Ig class to the descendant Ig class. These results suggest that CSR does not occur preferentially in a given B-cell population with limited IGHV, IGHD, or GHJ; therefore, CSR would not perturb the Ig repertoire under physiological conditions. The length of CDR3 creating an antigen-binding region shortened during the class-switching process from IgM to the other isotypes or subclasses. This is similar with the CDR3 shortening observed in TCR during the thymocyte development (27, 28). IgH-CDR3 has been reported to be shorter in highly mutated Ig than non-mutated Ig (29). During maturation of B cells, a small portion of IgM with shorter CDR3 will be preferentially selected to switch to IgG. Antibodies capable of effective binding to antigen are suggested to favor shorter length of CDR3.

There were no differences in the SHM levels among IgG1, IgG2, and IgG3. It has been reported that there were higher levels of mutation in IgG2 than in other IgG subtypes and in IgA2 than in IgA1 (30). However, here, we did not observe such a difference in SHM; instead, similar levels of SHM were found in all IgG subclasses except IgG4. This was true for subclass-specific sequences, but not for sequences shared between subclasses. Importantly, the subclass-specific sequences had higher levels of SHM than the shared sequences. IgM first undergoes CSR to switch to IgG3, and then switches to IgG1 and IgG2 to obtain higher antigen affinity. In fact, it has been reported that IgG3 deficiency was frequently associated with a decrease in other IgG subclasses (31). Despite switching from IgG3 to downstream IgGs, IgG1 and IgG2 did not show high levels of SHM. These results suggest that CSR occurs sequentially over multiple subclasses with little emerging SHM and, thereafter, SHMs increasingly accumulate in subclass-specific lineages.

IgG4 is mainly produced by switching from IgG2. It has been reported that low concentrations of IgG2 frequently occur in association with a deficiency in IgG4 (32). We also found that IgG4-specific sequences had higher SHM levels than the other subclasses; however, there was no such difference in the reads shared among IgG3, IgG1, IgG2, and IgG4. Similarly, IgA1-specific or IgA2-specific sequences had higher levels of SHM than the clones shared between IgA1 and IgA2. Interestingly, Ig clones were frequently shared among multiple isotypes including IgG, IgA, and IgE. These results suggest that the shared clones undergo sequential transitional from the ancestral Ig subclass to the descendant Ig class without SHM and subsequent accumulation of abundant SHMs in the Ig-specific lineage.

Recently, Horns et al. have reported a large scale of antibody repertoire sequencing with subclass resolution in twin cohort [Ref. (33), p. 3058]. They have described sequential CSR from IgM to proximal classes (IgG3, IgG1, or IgA1) and then to distal classes (IgG2, IgG4, or IgA2) in healthy twin samples. In consistent with the report, we have also observed higher rate of shared clones between combinations of the proximal and the distal classes (e.g., IgG1 and IgG2, IgA1, and IgA2).

Although both SHM and CSR require the activity of AID (34, 35), B cells proliferate vigorously in lymphoid tissues upon activation by antigen stimulation to undergo SHM and CSR. The accumulation of SHMs is accelerated to increase antigen affinity, playing a critical role in the antibody response against viral and bacterial pathogens including HIV, influenza, and *Streptococcus pneumoniae* (36–38). CSR is also driven by stimulation with pathogenic antigens or environmental stimuli. However, unlike antigen stimulation, SHM might be inactive in the normal setting of healthy individuals. There is a need to perform further study on the differential accumulation of SHMs in B cells under antigen stimulation or in disease conditions.

In conclusion, NGS-based antibody repertoire analysis provided insight into Ig class switching with SHM under physiological conditions, where CSR is suggested to occur more quickly than SHMs accumulate. This repertoire analysis should provide deep insight into antibody maturation and boost our understanding of immune reactions.

## Ethics Statement

After obtaining written informed consent, whole blood samples were collected from 12 healthy individuals. This study was approved by the ethics committees of the Clinical Research Center for Rheumatology and Allergy, Sagamihara National Hospital, National Hospital Organization.

## Author Contributions

KK, HY, and HA carried out the experiments. TS developed sequence analysis software. TM designed the study, performed data analysis, and wrote the manuscript. RS designed the study. All the authors read and approved the final manuscript.

## Conflict of Interest Statement

The authors declare that the research was conducted in the absence of any commercial or financial relationships that could be construed as a potential conflict of interest.

## Abbreviations

TCR, T-cell receptor; BCR, B-cell receptor; Ig, immunoglobulin; V, variable; D, diversity; J, joining; C, constant; CDR, complementarity-determining region; CSR, class-switch recombination; SHM, somatic hypermutation; PBMC, peripheral blood mononuclear cell.
