# Supplementary material for: Different Somatic Hypermutation Levels among Antibody Subclasses Disclosed by a New Next-Generation Sequencing-Based Antibody Repertoire Analysis
Source: Front Immunol. 2017 May 3;8:389. doi: 10.3389/fimmu.2017.00389 (PMC5413556; doi:10.3389/fimmu.2017.00389)
Supplement: Supplementary file 1 [file Data_Sheet_1.PDF]

Supplemental Table 1

Table 1 Numbers of total reads and unique reads obtained in this study

Individuals	Total Reads (Unique Reads)				
	IgM	IgD	IgG	IgA	IgE
IV01	136772	103259	147336	128894	17331
	(36897)	(16332)	(28790)	(24405)	(817)
IV02	131760	56232	94980	120408	3327
	(47182)	(15310)	(23995)	(25477)	(277)
IV03	143158	85372	112464	99473	19111
	(38411)	(13393)	(32655)	(33131)	(933)
IV04	156034	182340	137568	262108	303
	(21254)	(16519)	(17263)	(30897)	(253)
IV05	120871	64045	112285	87882	12731
	(17093)	(7314)	(14518)	(13416)	(590)
IV06	139027	154276	101050	104090	2632
	(32065)	(20066)	(23861)	(20353)	(264)
IV07	143075	78600	68265	9575	224
	(14918)	(7658)	(14196)	(5367)	(164)
IV08	156775	65743	39566	80163	3739
	(23675)	(10065)	(12085)	(17062)	(242)
IV09	68825	104478	131653	122880	148
	(8243)	(8926)	(17494)	(17245)	(132)
IV10	62122	77989	123714	113503	12895
	(18774)	(12872)	(21715)	(18076)	(622)
IV11	64804	277688	94651	138870	2469
	(29486)	(42714)	(19623)	(26936)	(360)
IV12	58392	76210	112663	138275	14967
	(35511)	(20626)	(31783)	(41428)	(915)

Supplemental Table 2

Table 2 Frequencies (%) of in-frame reads among total reads and unique reads

Individuals	Total Reads (Unique Reads)				
	IgM	IgD	IgG	IgA	IgE
IV01	96.8	96.4	98.7	98.5	99.0
	(94.47)	(91.16)	(95.95)	(95.05)	(87.39)
IV02	96.5	96.1	98.4	98.6	96.7
	(94.81)	(93.27)	(95.67)	(95.57)	(86.64)
IV03	96.4	96.0	98.6	98.8	98.8
	(94.02)	(90.27)	(96.63)	(96.79)	(88.00)
IV04	97.4	96.2	99.1	98.5	87.1
	(92.23)	(88.35)	(94.91)	(93.10)	(84.58)
IV05	97.1	96.1	98.4	97.8	99.2
	(91.86)	(88.30)	(94.21)	(92.88)	(87.29)
IV06	96.5	96.6	98.8	98.4	98.3
	(93.38)	(89.61)	(96.12)	(94.87)	(85.61)
IV07	98.1	97.8	98.8	98.7	92.0
	(91.68)	(89.15)	(96.19)	(98.02)	(89.02)
IV08	97.4	98.4	98.9	99.1	99.5
	(93.02)	(93.32)	(97.24)	(96.44)	(93.39)
IV09	96.4	98.9	98.5	98.7	90.5
	(90.79)	(91.51)	(94.14)	(93.82)	(89.39)
IV10	96.7	96.0	98.8	98.8	99.0
	(94.57)	(90.38)	(95.38)	(94.74)	(85.85)
IV11	96.7	95.9	98.6	98.5	97.4
	(95.56)	(90.26)	(95.73)	(95.19)	(83.06)
IV12	96.3	95.6	98.5	98.7	98.0
	(95.70)	(92.51)	(96.16)	(96.53)	(90.05)

Supplemental Table 3. Number of clones shared among multiple subclasses

Shared Ig subclasses	Number of clones
IgM --- ---- ---- ---- ---- ---- --- ----	299620
--- IgD ---- ---- ---- ---- ---- --- ----	172078
--- --- ---- ---- IgA1 ---- ---- --- ----	105239
--- --- ---- ---- ---- IgG2 ---- --- ----	71306
--- --- ---- IgG1 ---- ---- ---- --- ----	67097
--- --- ---- ---- ---- ---- ---- --- IGA2	58145
--- --- ---- ---- IGA1 ---- ---- --- IGA2	42126
--- --- ---- IgG1 ---- IgG2 ---- --- ----	23216
--- --- IgG3 ---- ---- ---- ---- --- ----	7986
--- --- IgG3 IgG1 ---- IgG2 ---- --- ----	6789
--- --- ---- ---- ---- ---- ---- IgE ----	3615
--- --- ---- ---- ---- ---- IgG4 --- ----	2565
--- --- IgG3 ---- ---- IgG2 ---- --- ----	1746
--- --- IgG3 IgG1 ---- ---- ---- --- ----	1484
--- --- ---- IgG1 IGA1 IgG2 ---- --- IGA2	1060
IgM IgD ---- ---- ---- ---- ---- --- ----	1022
--- --- IgG3 IgG1 ---- IgG2 IgG4 --- ----	816
--- --- IgG3 IgG1 IGA1 IgG2 ---- --- IGA2	782
--- --- ---- IgG1 ---- IgG2 IgG4 --- ----	770
--- --- ---- ---- ---- IgG2 ---- --- IGA2	744
IgM --- ---- ---- IgA1 ---- ---- --- IgA2	707
IgM --- ---- ---- IgA1 ---- ---- --- ----	555
--- --- ---- ---- IGA1 IgG2 ---- --- IGA2	544
--- --- ---- ---- IgA1 IgG2 ---- --- ----	485
--- --- ---- IgG1 IgA1 IgG2 ---- --- ----	431
--- --- ---- IgG1 ---- IgG2 ---- --- IGA2	384
--- --- ---- IgG1 IgA1 ---- ---- --- ----	371
--- --- ---- IgG1 ---- ---- IgG4 --- ----	312
--- --- ---- IgG1 IGA1 ---- ---- --- IGA2	278
--- --- ---- IgG1 ---- ---- ---- --- IGA2	221
IgM --- ---- ---- ---- ---- ---- --- IgA2	215
--- --- ---- ---- ---- IgG2 IgG4 --- ----	193
--- --- IgG3 IgG1 ---- IgG2 ---- --- IGA2	175
--- --- IgG3 IgG1 IgA1 IgG2 ---- --- ----	150
--- --- IgG3 IgG1 IGA1 IgG2 IgG4 --- IGA2	147
--- --- IgG3 IgG1 ---- ---- IgG4 --- ----	127
IgM --- ---- IgG1 ---- IgG2 ---- --- ----	114
--- --- IgG3 ---- ---- ---- IgG4 --- ----	111
IgM --- ---- ---- ---- IgG2 ---- --- ----	89
--- IgD ---- ---- IGA1 ---- ---- --- IGA2	88
IgM --- ---- IgG1 ---- ---- ---- --- ----	85
--- --- IgG3 ---- IGA1 IgG2 ---- --- IGA2	84
--- IgD ---- ---- IgA1 ---- ---- --- ----	78
IgM --- ---- IgG1 IgA1 IgG2 ---- --- IgA2	69
IgM --- IgG3 IgG1 IgA1 IgG2 ---- --- IgA2	68
--- --- IgG3 ---- ---- IgG2 IgG4 --- ----	67
--- --- IgG3 ---- ---- IgG2 ---- --- IGA2	49
--- --- IgG3 IgG1 IGA1 ---- ---- --- IGA2	47
--- --- IgG3 ---- IgA1 ---- ---- --- ----	46

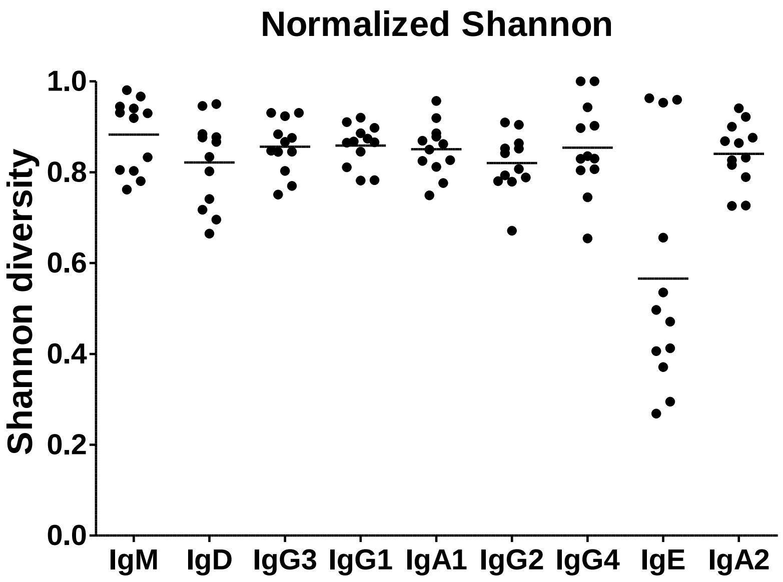

Supplemental Figure 1

Diversity of Ig subclasses

Normalized Shannon–Weaver index was calculated with the number of sequence reads from respective Ig subclasses, as described in the Materials and Methods. Each dot represents a healthy individual (n=12). There were no differences in the diversity among IgM, IgD, IgG3, IgG1, IgA1, IgG2, IgG4 and IgA2, while the diversity in IgE was lower than in the others.

**IgM**	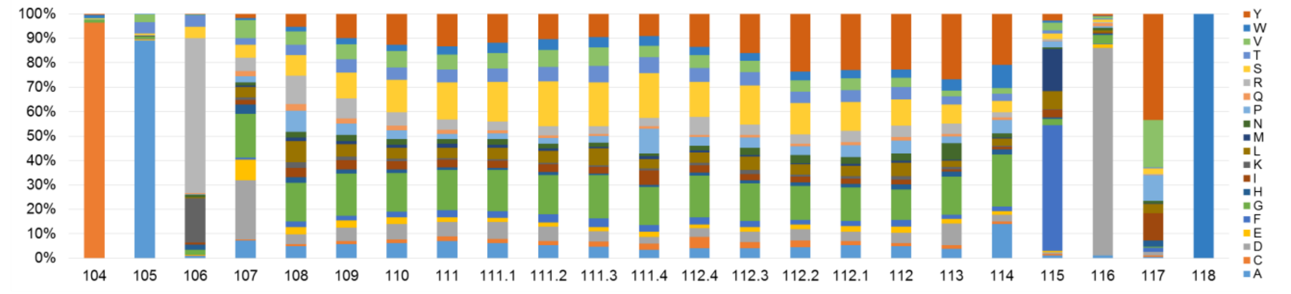
**IgD**	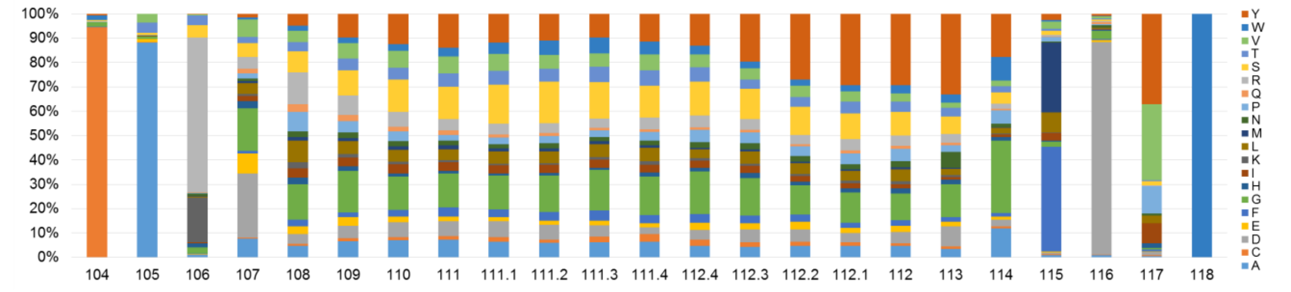
**IgG3**	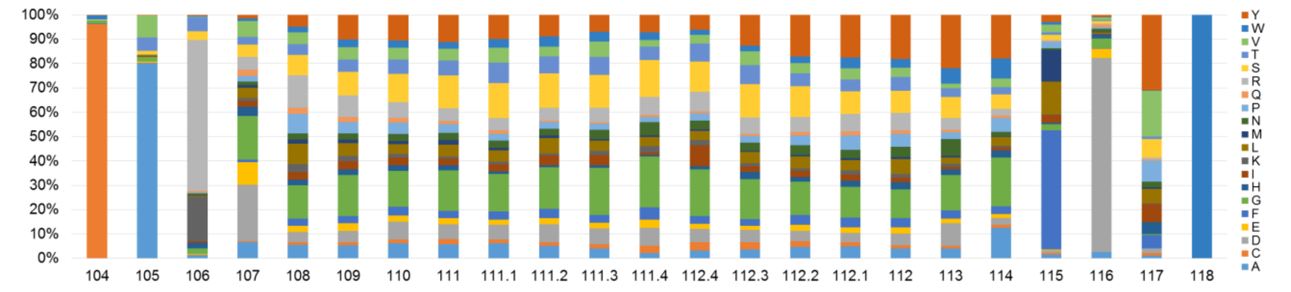
**IgG1**	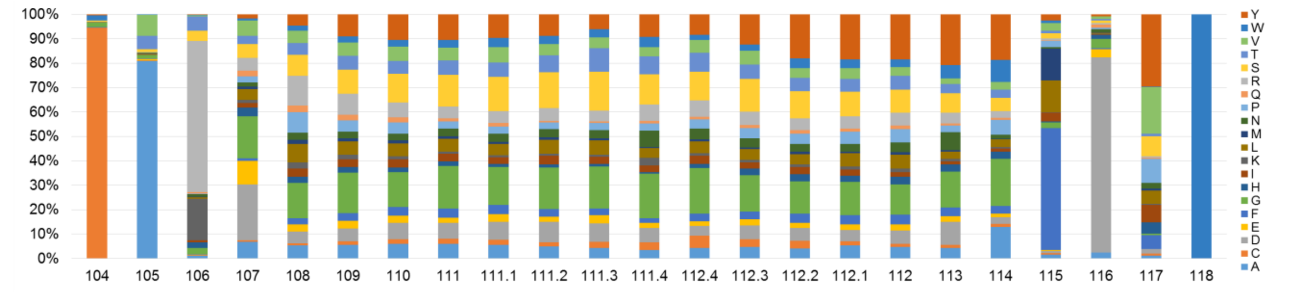
**IgA1**	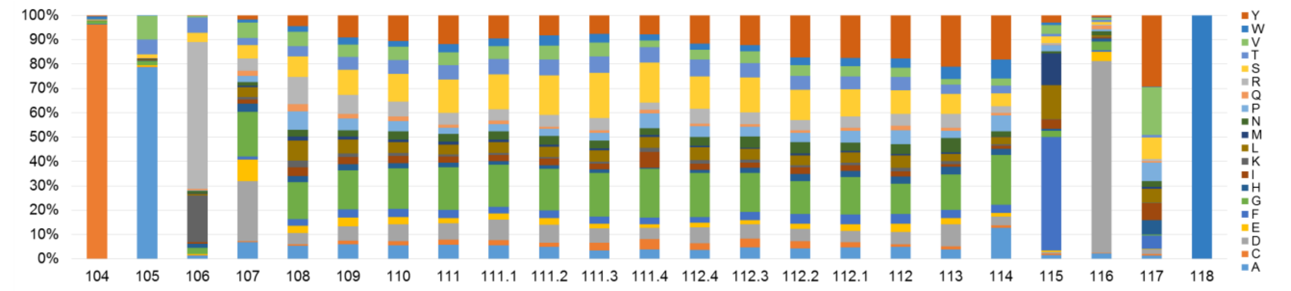
**IgG2**	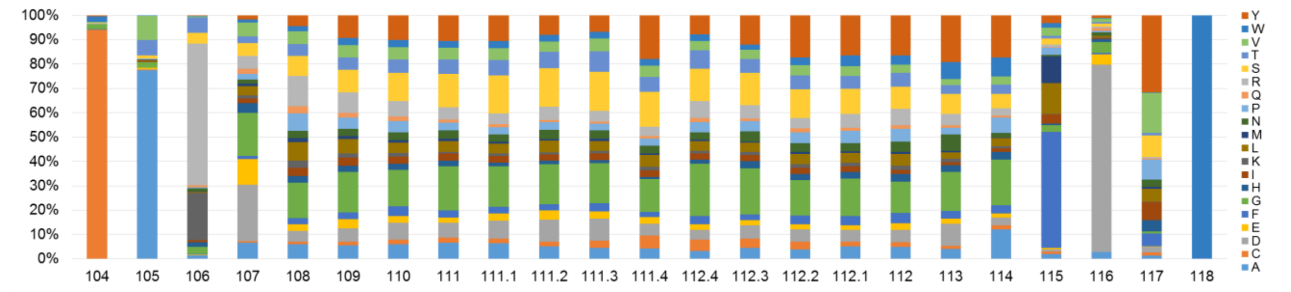

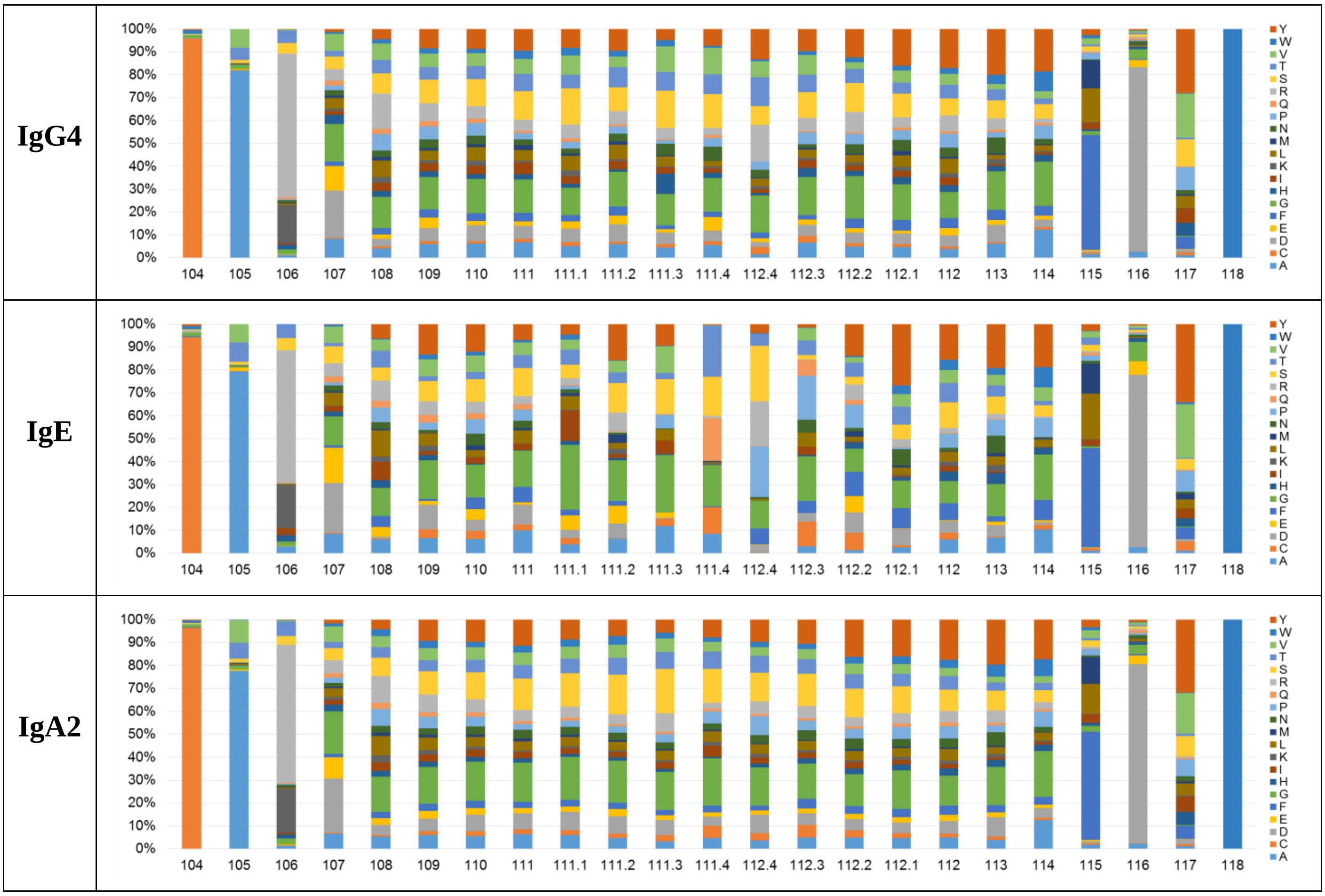

Supplemental Figure 2

Comparison of amino acid composition of CDR3 among Ig isotypes and subclasses

Percentage frequencies of amino acid composition were calculated at each position from anchored 104 Cys to 118 Trp. Amino acid was indicated by a single-letter amino acid code in different color. Position of CDR3 amino acid was numbered according to CDR3-IMGT nomenclature.

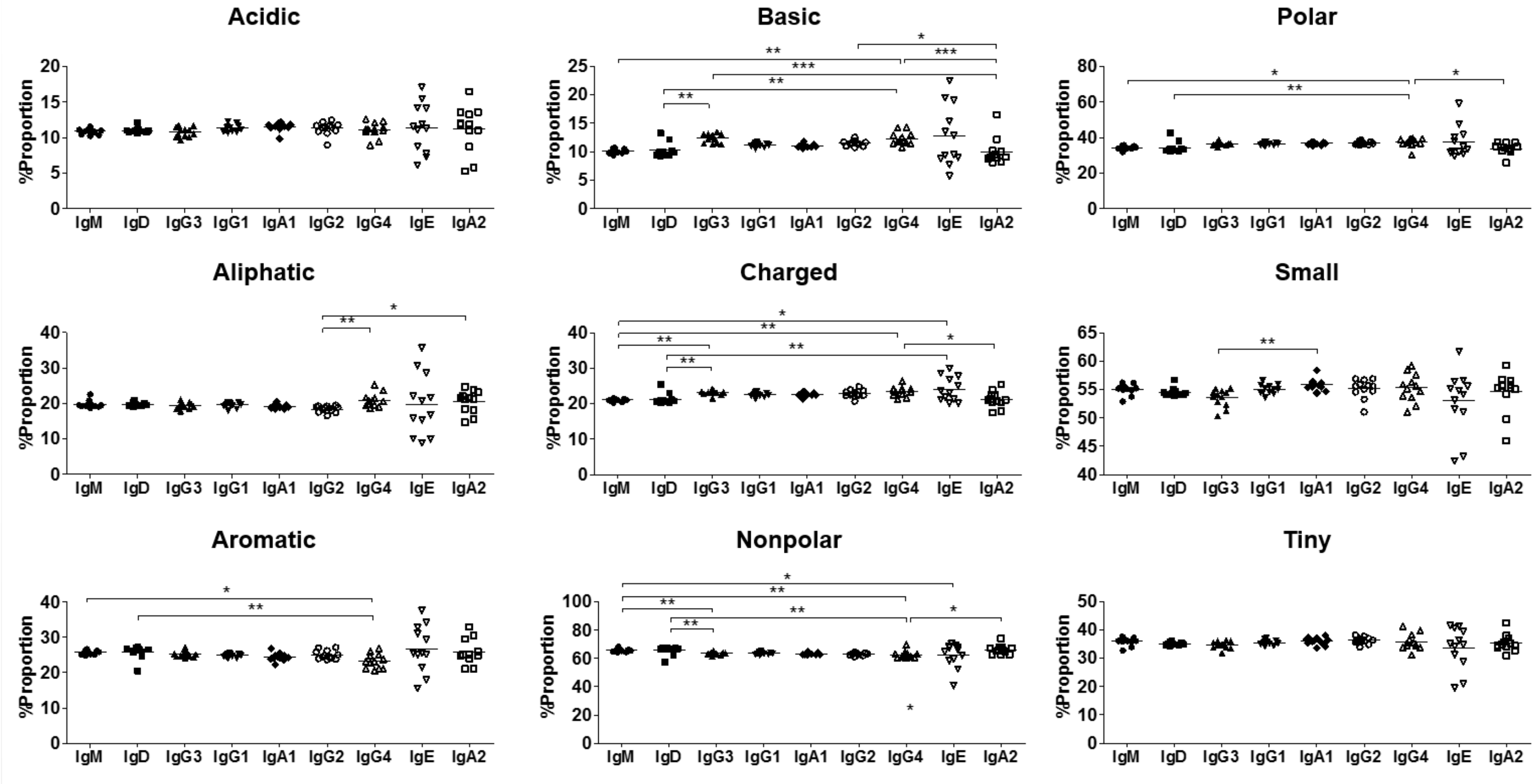

Supplemental Figure 3

Comparison of physicochemical properties of CDR3 amino acid among Ig isotypes and subclasses

Percentage frequencies of amino acid residues classified by structural and functional properties in CDR3 sequence were shown. Each dot indicates each healthy individuals (n=12). Amino acid were classified into Tiny (A, C, G, S, T), Small (A, B, C, D, G, N, P, S, T, V), Aliphatic (A, I, L, V), Aromatic (F, H, W, Y), Non-polar (A, C, F, G, I, L, M, P, V, W, Y), Polar (D, E, H, K, N, Q, R, S, T), Charged (B, D, E, H, K, R, Z), Basic (H, K, R), and Acidic (B, D, E, Z). B: D/N, Z: Q/E. Statistical significance was tested with Kruskal-Wallis test followed by Dunn's post hoc test. *P < 0.05, **P < 0.01, ***P < 0.001
